# Case Report: B Lymphocyte Disorders Under COVID-19 Inflammatory Pressure

**DOI:** 10.3389/fonc.2020.582901

**Published:** 2021-01-25

**Authors:** Gloria Taliani, Elena Follini, Lorenzo Guglielmetti, Patrizia Bernuzzi, Alberto Faggi, Patrizia Ferrante, Elisa Fronti, Laura Gerna, Maria Cristina Leoni, Franco Paolillo, Giovanna Ratti, Alessandro Ruggieri, Caterina Valdatta, Alessandra Donisi, Adriano Zangrandi, Lara Pochintesta, Carlo Moroni, Daria Sacchini, Daniele Vallisa, Mauro Codeluppi, the COVID-Piacenza Group, Daniela Aschieri

**Affiliations:** ^1^Infectious Diseases Unit, “Guglielmo da Saliceto” Piacenza Hospital, Piacenza, Italy; ^2^Anti-COVID-19 Task Force of the Italian Civil Protection, Rome, Italy; ^3^Infectious and Tropical Disease Unit, Department of Translational and Precision Medicine, Sapienza University of Rome, Rome, Italy; ^4^Hematology and Bone Marrow Transplant (BMT) Unit, “Guglielmo da Saliceto” Piacenza Hospital, Piacenza, Italy; ^5^Sorbonne Université, INSERM, U1135, Centre d’Immunologie et des Maladies Infectieuses, Cimi-Paris, équipe 13, Paris, France; ^6^APHP, Groupe Hospitalier Universitaire Sorbonne Université, Hôpital Pitié-Salpêtrière, Centre National de Référence des Mycobactéries et de la Résistance des Mycobactéries aux Antituberculeux, Paris, France; ^7^Institute for Cross-Disciplinary Physics and Complex Systems IFISC (UIB-CSIC), Campus Universitat Illes Balears, Palma de Mallorca, Spain; ^8^Migration Health Unit, Primary Health Care Department, “Guglielmo da Saliceto” Piacenza Hospital, Piacenza, Italy; ^9^Pathology Unit, “Guglielmo da Saliceto” Piacenza Hospital, Piacenza, Italy

**Keywords:** COVID-19, B-cell lineage malignancies, reactivation, autoimmune hemolytic anemia, multiple myeloma, acute lymphoblastic leukemia

## Abstract

Severe acute respiratory syndrome coronavirus-2 (SARS-CoV-2) infects humans through the angiotensin converting enzyme-2 (ACE-2) receptor expressed on many cells, including lymphocytes. In Covid-19 patients IL-6 is overexpressed, and hyperactivated plasmacytoid lymphocytes are detected in peripheral blood film. We hypothesize that, due to the unpredictable interaction between the new virus and the B cell lineage of infected patients, a cascade of out of control events can ensue, capable of determining unexpected pathologic disorders involving such lineage. Here we report two cases of autoimmune hemolytic anemia (AIHA) and two cases of B-cell hematological malignancies developed or reactivated during acute SARS-CoV-2 infection. The temporal relationship of the events may suggest a potential causal relationship between SARS-CoV-2 infection and the hematopoietic disorders. We suggest that special attention should be paid to COVID-19 patients with underlining B cell lineage disorders.

## Introduction

COVID-19 is a rapidly spreading disease due to the SARS-CoV-2 virus that infects humans through the angiotensin converting enzyme (ACE-2) receptor.

In patients with COVID-19, some pro-inflammatory cytokines, such as Interleukin-(IL-)6, IL-1β, and tumor necrosis factor-α (TNF-α), are frequently released by lung epithelial cells as well as by lymphocytes (1). Among these, IL-6 is one of the most important, and it is involved in many aspects of the COVID-19 clinical picture. IL-6 is a cytokine with pleiotropic biological functions, ranging from pro-inflammatory effects in acute innate responses to anti-inflammatory activities (2). In addition, it is capable of modulating the production of hematopoietic cytokines such as granulocyte-colony stimulating factor (G-CSF) and granulocyte-macrophage colony stimulating factor (GM-CSF), that stimulate the expansion of myeloid lineages (3). The ACE2 receptor is expressed also on the surface of lymphocytes, thus SARS-CoV-2 may infect such cells. Indeed, in COVID-19 patients, lymphopenia is one of the most commonly observed features, probably due to lymphocytes induced apoptosis, and it seems to be associated with disease severity and mortality. Patients with severe illness cases show lower number of total lymphocytes and B cells than patients with mild disease cases, and recovery is associated with a significant increase in total lymphocytes (4), and the same peripheral lymphocyte decrease similar lymphocytopenia has been described also in patients with pneumonia caused by SARS-Cov and MERS-Cov (5). Moreover, corticosteroid treatment, which usually decreases the lymphocytic count, induces a significant increase of lymphocytes in COVID-19 patients, indicating that corticosteroid anti-inflammatory properties might outweighed its lympho-lytic effects (6, 7). Interestingly, in Covid-19 patients, lympho-plasmacytoid lymphocytes are observed, similar to the reactive lymphocytes seen in dengue fever and in several B-cell non-Hodgkin lymphomas (8) indicating the hyperactivated status of peripheral CD4 and CD8 T cells, which is associated to an increased concentration of highly proinflammatory CCR6+ Th17 in CD4 T cells (9).

The other typical feature of SARS-Cov-2 infection is overexpression of IL-6 that, under control of the enhancer of the gene encoding the human immunoglobulin chain, is capable of inducing plasmacytosis and may primarily serve as a factor driving the development and/or survival of plasma cells, and isotype switching (8). Therefore, we hypothesize that, due to the unpredictable interaction between this new virus and the B cell lineage of infected patients, a cascade of out of control events can ensue determining some unexpected pathologic conditions such as the progression reactivation of hematological malignancies or the development of autoimmune disorders. We report four cases of hematopoietic disorders involving the B cell lineage developed during SARS-CoV-2 infection.

## Methods

RT-PCR for Sars-CoV-2 was performed on nasal swabs by Abbott RealTi*m*e SARS-C0V-2 assay (Abbott Laboratories, Chicago, Ill).

Hemolysis tests included complete blood count, reticulocyte count, LDH, total and direct bilirubin, haptoglobin, direct antiglobulin test (DAT), and indirect antiglobulin test (IAT).

Immunohistochemical staining was performed using the Ventana (Roche Diagnostics, Basel, Switzerland) automated immune-stainer with a polymer-based detection system. Primary antibodies employed were from Ventana (Roche Diagnostics, Basel, Switzerland); a mouse monoclonal primary antibody (Clone: B-A38) was used for CD138 immunostaining.

### Antiviral Treatment

Standard antiviral therapy with hydroxychloroquine (loading 400 mg/bid, then 200 mg/bid/6days) and darunavir-cobicistat 800/150 mg for 7 days (patients #1, #2, and #4) or azithromycin 500 mg/day for 7 days (patient #3) was employed according to the hospital guidelines existing at that time.

### Ethics

Written informed consent was obtained from the individuals for the publication of any potentially identifiable images or data included in this article. Patients perspective was given our most careful attention. All subjects were treated with dignity, compassion, and respect, and coordinated care and support were offered with personalized support, and treatment.

### Clinical Cases

In **Table 1** are reported the demographic, clinical, therapeutic characteristics, and outcome of the four enrolled patients, while in **Table 2** are reported the most significant biochemical data observed during hospitalization, the highest values observed for inflammatory parameters, and the lowest values of hemoglobin and albumin recorded during hospital stay.

**Table 1:** Demographic, clinical, therapeutic characteristics, and outcome of the four patients.

	Sex	Age	Disease	Stage/risk	Therapy	Disease-status before COVID-19 infection	Covid-19 Outcome
**Pt 1**	F	72	Warm AIHA	NA	Steroids, rituximab x 4	Remission	Recovery
**Pt 2**	M	56	Warm AIHA	NA	Steroids	Not present	Recovery
**Pt 3**	F	63	IgG k MM	ISS I	VTD x 4	PR	Death
**Pt 4**	F	59	Ph+ ALL	High	Hyper-CVAD + imatinib, dasatinib, MUD-HSCT, ponatibin, inotuzumab-ozogamicin × 5	CR, MRD 0,005	Recovery

AIHA, autoimmune hemolytic anemia; MM, multiple myeloma; Ph+ ALL, Philadelphia-positive acute lymphoblastic leukemia; VTD, velcade-thaldomide-dexamethasone; Hyper-CVAD, cyclophosphamide, vincristine, adriamicine, dexamethasone; MUD-HSCT, matched unrelated donor hematopoietic stem cell transplantation; NA, not applicable; ISS, international staging system; PR, partial remission; CR, complete remission; MRD, molecular residual disease.

**Table 2:** Laboratory values of the four patients obtained during hospitalization.

	Case 1		Case 2		Case 3	Case 4
	First admission	Second admission	First admission	Second admission	First admission	First admission
**Values at hospital admission**						
**White Blood Cells/mm^3^**	8,260	8,520	5,070	4,640	9,740	5,300
**Lymphocytes cells/mm^3^**	1,880	1,860	1,250	960	1,700	1,700
**Eosinophyls cells/mm^3^**	0	0	0	40	0	70
**Hemoglobin (g/dl)**	7.3	7.6	11.9	5.9	9.6	14.9
**Platelets ×10^3^/mm^3^**	411	192	308	272	138	147
**LDH (U/L)**	389	310	782	499	464	242
**CRP (mg/dl)**	5.41	0.71	6.48	0.51	21.11	0.37
**Gamma globulin (g/dl)**	0.40	0.31	1.16	1.19	0.49	0.98
**Higer values recorded during hospitalization**						
**Bilirubin total/direct mg/dl**	2.56/0.44	1.83/0.46	1.04/0.2	3.16/0.52	1.07/0.4	1.3/0.2
**Reticulocytes/1,000 RBC**	88	106	ND	146.5	ND	ND
**D-dimer (ng/ml)**	NA	746	679	1033	13585	1138
**IL6 (pg/ml)**	23.5	30.5	9.6	31.9	422.8	24.2
**Ferritin (ng/ml)**	1,048	487	1,909	620	7,080	1,873
**LDH (U/L)**	493	584	782	499	1455	519
**CRP (mg/dl)**	9.73	0.71	9.51	0.51	28.74	0.37
**Lower values recorded during hospitalization**						
**Albumin (g/dl)**	NA	3.2	NA	4	1.7	4.53
**Hemoglobin (g/dl)**	6.6	7.6	9.1	5.9	7	11.2
**Haptoglobin (mg/dl)**	<1	<1	ND	<1	ND	ND
**Lymphocytes (cells/mm^3^)**	470	1,100	1,250	700	510	1,270
**Vital signs at admission**						
**Temperature (°C)**	38.2	36	37.7	36.5	38.3	37.7
**Blood pressure (mmHg)**	135/80	120/60	120/85	140/80	145/70	145/90
**Sp02 (%)**	96	97	93	99	99	97
**Heart rate (bpm)**	75	115	76	81	100	99

CRP, C Reactive Protein; LDH, Lactic Dehydrogenase; ND, not done; RBC, Red Blood Cells.

### Patient 1

A 72 years-old woman referred to Piacenza Hospital in September 2019 due to fatigue and dyspnea. Complete blood count (CBC) showed severe macrocytic anemia with increased reticulocyte count (Hb 8.3 g/dl, MCV 97,2 fL, reticulocyte 180/1,000 RBC) and abnormal hemolysis tests (LDH 445 U/L, total/direct bilirubin 2.54/0.32 mg/dl) with low haptoglobin levels (<1 mg/dl). Direct antiglobulin test (DAT) detected IgG and C3. Bone marrow biopsy excluded an underlying lymphoproliferative disease and autoimmunity tests were negative. Warm autoimmune hemolytic anemia (AIHA) was diagnosed and, after failure of steroid treatment, four successful courses of iv Rituximab 375 mg/m^2^/week were undertaken. Hemoglobin levels promptly rose up to 11 g/dl and remained stable after steroid tapering to prednisone 5 mg/day. On March 13, 2020 the patient presented with fever, dyspnea, hypoxia (SaO2 94%), diarrhea and marked fatigue: she underwent a RT-PCR assay for Sars-CoV-2 from upper respiratory-tract swab which resulted positive and she performed a CT scan showing bilateral interstitial pneumonia leaving 75% visually well aerated lung (%V-WAL) (9). After admission, the hemolytic process flared, causing severe symptomatic anemia (Hb 6.6 g/dl, MCV 90 fL, reticulocyte 88/1,000 RBC, LDH 430 U/L, total and direct bilirubin 1.56/0,44 mg/dl, haptoglobin <1 mg/dl) (**Table 1**). Virological investigations for EBV, CMV, VZV, Parvovirus B-19, and HIV resulted negative. Therapy with hydroxychloroquine (loading 400 mg/bid, then 200 mg/bid/6days) and darunavir-cobicistat 800/150 mg for 7 days was started associated with prednisone 1 mg/kg. The patient was maintained on low-flow oxygen therapy for 7 days and received two red-blood-cell Units. On 14^th^ April, after progressive tapering of steroid treatment, Sars-CoV-2 nasopharyngeal swab was negative, fever and dyspnea were absent, hemoglobin was 12.5 g/dl, reticulocyte count was 17.9/1,000 RBC, and she was discharged.

**Figure 1:**
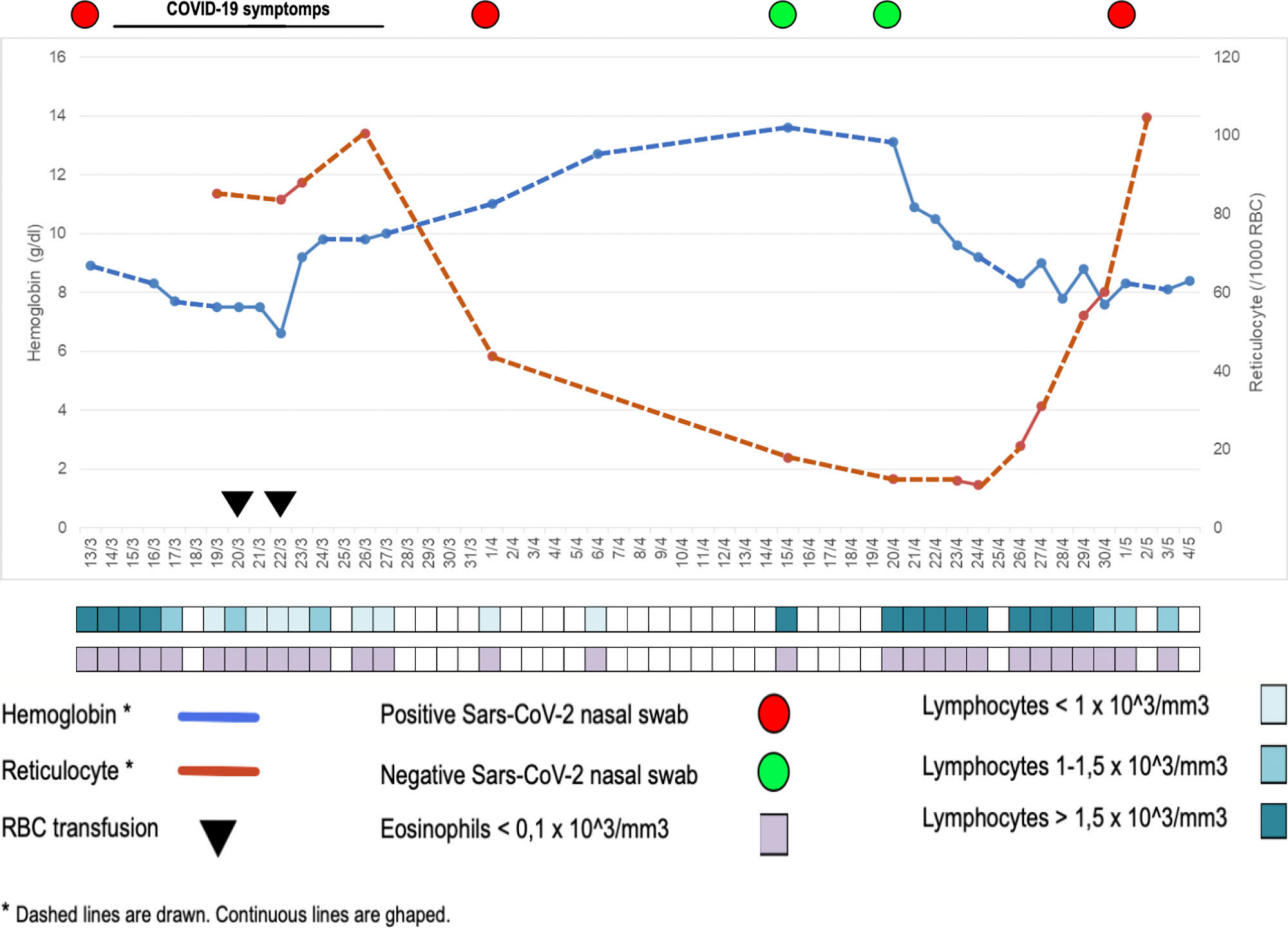
Hematologic, clinical, and virological data of patient #1, indicating the concomitant occurrence of hemolysis and virus SARS-CoV-2 detection in nasal swab.

On April 26^st^ she was re-admitted to hospital for a relapse of hemolytic anemia, with increasing LDH (534 UI/L), transaminases (GOT 310 UI/L, GPT 486 UI/L), decreasing hemoglobin (8.3 g/dl) levels and decreasing lymphocyte (830/μl) and eosinophil (0/μl) count (**Figure 1**). An immunosuppressive treatment cycle with azathioprine was started. The patient was asymptomatic for respiratory symptoms, but her Sars-CoV-2 nasal swab turned-out to be again positive.

### Patient 2

A 56 years-old man with silent previous unremarkable medical history was admitted to hospital on March 21^st^ due to fever >38°C, dyspnea, and vomiting. His upper respiratory tract Sars-Cov-2 sample was positive, and chest CT scan showed mild COVID-19 bilateral interstitial pneumonia leaving 95% visually well aerated lung (%V-WAL) (9). He was hospitalized and treated with hydroxychloroquine (loading 400 mg/bid, then 200 mg/bid/6days) and darunavir-cobicistat 800/150 mg for 7 days. Antiviral therapy associated with low-flow oxygen maintenance led to a rapid clinical benefit. During hospitalization he developed a mild normocytic anemia (Hb 9.9 g/dl, MCV 97.2 fL) which was deemed secondary to chronic inflammation, as shown by high inflammatory indexes (PLT 469,000/mm^3^, ferritin 1,909 ng/ml, D-dimer 679 ng/ml, CRP 9.51 mg/dl). He was discharged on March 30^th^. Due to profound asthenia, on April 21^st^ he underwent blood testing that showed worsening anemia (Hb 8.1 g/dl), and on April 28^th^ repeated Sars-CoV-2 nasal swab that tested positive. On May 6^th^ he presented to emergency room for marked fatigue. Severe macrocytic anemia (Hb 5.9g/dl, MCV 125.4 fL), raised reticulocyte count (146.5/1,000 RBC) and lymphopenia (L 960/mm^3^, **Table 2**) were found and hemolysis tests showed: total/direct bilirubin 3.16 mg/dl and 0.52 mg/dl, LDH 499 U/L, haptoglobin < 1mg/dl. Direct antiglobulin test was positive (IgG +++). Virological investigations for EBV, CMV, VZV, Parvovirus B-19, and HIV resulted negative. Prednisone 1 mg/kg and transfusion of two packed red blood cells (pRBCs) have restored hemoglobin levels and clinical conditions. Testing for autoimmune or infective underlying causes of AIHA (HBV, HCV, ANA) was negative, and no history of recent drug exposure was reported. We therefore hypothesized that COVID-19 infection might have triggered AIHA.

### Patient 3

**Figure 2:**
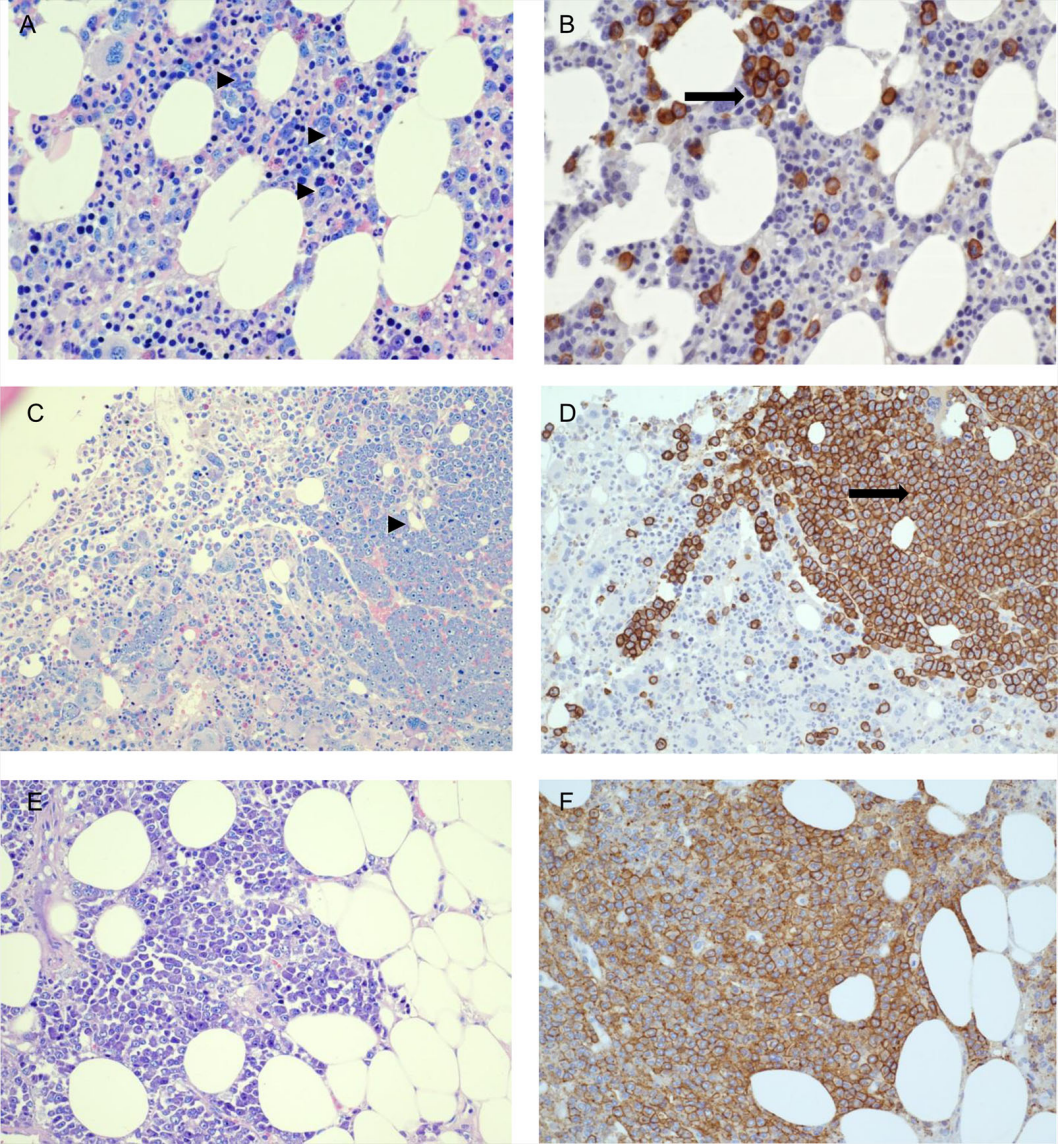
Patient #3. Bone marrow biopsy performed on November, 2019 when multiple myeloma was diagnosed; bone marrow and skin biopsies performed on March, 2020 at the time of hospital admission with COVID-19. **(A, B)** Patient-3 bone marrow biopsy at diagnosis (11/11/2019), showing clonal plasma-cells scattered or in small groups, accounting for 15% of infiltrate (Giemsa staining: ►; CD138 immunohistochemical staining: ➞) **(C, D)** Second bone marrow biopsy post-acute COVID-19 infection (23/04/2020) showing abnormal and massive plasma-cellular infiltrate by Giemsa staining (right) (►) and CD138 immunohistochemical staining (➞), with immature morphology and evidence of angioinvasion. **(E, F)** Skin biopsy post COVID-19 infection (28/04/2020) showing massive plasma-cell infiltrate in subcutaneous fat. **(A, B)** 400× magnification. **(C–F)**: 200× magnification. **(A, C, E)**: Giemsa staining. **(B, D, F)**: CD138 immuno-histochemical staining.

A 63 years-old woman affected by IgG k monoclonal gammopathy of undetermined significance (MGUS) since 2016, came to our attention in November 2019 for back pain, increased monoclonal component (MC 2.8 g/dl), immune-paresis (IgG 3,828 mg/dl, IgA 30 mg/dl, IgM 31 mg/dl) and unbalanced free light chain (FLC) ratio (free kappa 398.83, free lambda 3.05, ratio k/lambda 130.7). Whole body magnetic resonance (WB-MRI), computed tomography (CT) and positron emission tomography (PET) scan evidenced multiple bone lesions and bone marrow examination detected 15% clonal plasma cells (**Figure 2**). Thus, MGUS had progressed to multiple myeloma, international staging system (ISS) stage I. The patient was eligible for autologous stem cell transplantation and started induction therapy with four cycles of bortezomib–thalidomide–dexamethasone (VTD) that induced a partial remission; therefore she was listed for stem cell harvest. On March 18^th^, 2020 she presented with mild fever and cough; her X-ray chest was normal, but Sars-CoV-2 upper respiratory tract swab resulted positive. hydroxychloroquine (loading 400 mg/bid, then 200 mg/bid/6days), and azithromycin 500 mg/day for 7 days were started and led to complete symptom regression. Nevertheless, on March 31^st^ she was admitted to Hospital for daily fever >39°C, abnormal C reactive protein (CRP 21.22 mg/dl). Serial blood cultures for bacteria and fungi were negative; transthoracic echo cardiac-US was negative for endocarditic vegetation and abdomen CT did not detect any abnormality, whole blood count dramatically worsened showing pancytopenia while inflammation indexes (CRP, IL6, ferritin) were significantly rising (**Table 2**). On April 15^th^ a bone marrow biopsy was performed which revealed a dramatic progression of the MM with 40% of abnormal plasma cells and evidence of hemo-phagocytosis and mild T-cell infiltrate (**Figure 2**). Few days later, her general conditions rapidly worsened because of acute respiratory failure, requesting a non-invasive ventilation (NIV). On April 18^th^ a further chest and abdomen CT scan demonstrated segmental pulmonary embolism, ground glass bilateral infiltrates, and multiple subcutaneous lesions. On April 20^th^ she was admitted to intensive care unit (ICU), was intubated, and mechanically ventilated. Bronchoalveolar lavage (BAL) was negative for respiratory viruses, cytomegalovirus, tuberculosis, pneumocystis jirovecii and galactomannan antigen. Skin biopsy detected extramedullary plasma cell infiltrates with evidence of immature phenotype (**Figure 2**). Patient’s clinical conditions worsened, multiorgan failure (MOF) ensued which caused her death on May 4^th^2020.

### Patient 4

A 55 years-old woman was diagnosed in 2016 with Philadelphia-positive (Ph+) acute lymphoblastic leukemia (ALL) and underwent a combined induction therapy with cyclophosphamide, vincristine, adriamicine, dexamethasone (Hyper-CVAD) and imatinib, followed by a second line with dasatinib in order to reach a deeper response, and finally on June 27, 2016 a 10/10 HLA matched-unrelated donor (MUD) allogeneic stem cell transplantation was performed. Eight months after HSCT she experienced a molecular relapse and acquired the T315I mutation, which was resistant also to ponatinib. Given the high burden of disease, in September 2017 she was enrolled in inotuzumab-ozogamicin compassionate use: up to five-cycle infusions associated with CNS prophylaxis led to a complete, deep and sustained molecular response. Until February 2020 her CSF-immunophenotypic and cytologic analyses remained negative for atypical lymphoid cells, while bone marrow aspirate revealed a BCR-ABL1/ABL transcript of 0.005. On April 8, 2020 she presented to hospital emergency room for mild fever and nausea. Her pharyngeal-nose swab tested positive for Sars-Cov-2, and she started a home treatment with hydroxychloroquine (loading 400 mg/bid, then 200 mg/bid/6days) and darunavir-cobicistat 800/150 mg for 7 days during which her conditions improved. On April 15 her symptoms worsened, and she was admitted to hospital with severe headache and fever. No signs of pulmonary involvement on chest HRCT-scan, and no ischemic or hemorrhagic lesions on brain CT-scan were detected. A lumbar puncture revealed 205 cells/mm^3^, with 57% lymphoblasts CD19+, CD34+, CD10+, CD20+/−, HLADR+, CD33+, CD117−. Sars-CoV-2 RNA was not found in cerebral-spinal fluid (CSF). Bone marrow aspirate confirmed a molecular relapse of her leukemia by detection BCR-ABL1/ABL transcript of 0.07. Weekly intrathecal chemotherapy with methotrexate 10 mg, cytarabine 40 mg, and dexamethasone 4 mg was started, and strict monitoring of her bone marrow disease has been undertaken to define the type and timing of salvage therapy.

## Discussion

Here we report four cases of hematopoietic disorders involving the B cell lineage in patients with proven, acute SARS-CoV-2 infection. Our cases suggest that COVID-19 may accelerate the course of malignant B cell diseases and induce or exacerbate autoreactive B cell mediated disease.

In COVID-19 patients, aberrant pathogenic T cells, inflammatory monocytes and other cells incite an inflammatory storm and induce a sharp and a great increase of IL-6 is observed (10), the levels of which significantly and directly correlate with the severity of the disease. Lung is the main target organ, but injuries in multiple organs, including spleen and bone marrow, have been reported. Therefore, a focus on the patients’ immune system may help to better understand SARS-CoV-2 infection. In particular, B lymphocytes and B-cell-derived-IL-6 seem to play a central role in SARS-CoV-2 infection and inflammatory disease, since in patients with agammaglobulinemia, who lack B cells, the COVID-19 course was characterized by mild symptoms, short duration, and favorable outcome without the need for treatment with an immune-modulating IL-6 blocking drug (11). Thus, we hypothesize that B lymphocytes actively participate to the development of the so-called cytokine storm by producing B-cell-derived-IL-6. On the other side, B lymphocytes may themselves be victims and may be affected by the consequences of the infection itself. inflammation they contribute to induce. IL-6 is capable of inducing a strong pro-inflammatory effect but also of controlling the immune responses, hematopoiesis, inflammation, and endocrine and nervous systems (12). It is well known that continuous production of IL-6 is implicated in the pathogenesis of auto-immune disorders, plasma-cell dyscrasias and many types of cancer (13). During initial interaction between antigen-primed autoreactive B cells and cognate CD4+ T cells, B cell-derived IL-6, which is essential to achieve the threshold of cytokine and costimulatory signals necessary for spontaneous germinal center (GC) formation, may lead to a break in B cell tolerance and induce systemic autoimmunity (14). Interestingly enough, in a mouse model, lack of B-cell-derived IL-6 is capable of abrogating spontaneous autoimmune GC formation, thus determining protection from systemic autoimmunity (14). The occurrence of hemolytic anemia has been recently reported in COVID-19 patients (15) and an erythrocyte membrane protein called ANK-1 has been found to share a putative immunogenic-antigenic epitope (amino acids LLLQY) with 100% identity with the spike surface glycoprotein of SARS-Cov-2 (12). Therefore, we speculate that the hyper-production of IL-6 may trigger the reappearance of the hemolytic anemia as we have observed in our patient #1, or induce it for the first time as occurred in patient #2. Moreover, a strict correlation between viral SARS-CoV-2 replication and hemolysis was observed in patient #1 (**Figure 1**). Indeed, after the first hemolytic event that developed when nasal swab was positive and after packed red blood cell transfusion, hemoglobin and reticulocytes had returned to normal and remained stable along with two negative nasal swabs, but a sharp hemoglobin level decrease and reticulocyte count increase occurred after the nasal swab returned positive. That is, the first hemolytic event occurred concomitantly with nasal swab positivity, resolution of hemolysis was accompanied by nasal swab negativity and, as expected, the subsequent viral reactivation was characterized by hemolysis reappearance. Thus, for the second time, the active Sars-CoV2 infection elicited an acute episode of AIHA.

Reappearance of swab positivity, associated to reappearance of symptoms in most of the patients, has been already described (16). Although some authors postulated the recurrence was actually attributable to a previous false PCR-negative result (17), the close temporal relationship between virus positivity and hemolytic crisis makes us lean towards a true virologic recurrence, probably from a body sanctuary, since the possibility of reinfection seems less likely (18). Moreover, such correspondence reinforces the causal association between viral replication and hemolysis. The same correspondence has been observed in patient #2, who presented his first episode of hemolytic anemia during SARS-Cov-2 infection, thus underlying the possible causal relationship between the two events.

Case #3 is a patient with partial remission of the MM disease waiting for harvesting her own bone marrow cells in view of auto-transplant. Interestingly, the early course of acute SARS-CoV-2 infection was very mild, without evidence of pulmonary involvement on CT scan. However, in two weeks her conditions worsened, she was readmitted due to uncontrolled high fever associated with abnormal plasma cells, mild T-cell infiltrate and phagocytosis in her bone marrow aspirate (**Figure 2**, panels C/D). Bone marrow microenvironment of MM patients may be affected by COVID-19 due to IL-6 over-production that has the ability to promote expansion and activation of T cells, differentiation of B cells and regulate the acute-phase response, thus creating an ideal microenvironment for oncogenesis (19) and inducing MM proliferation (20). Moreover, IL-6 produced by stromal cells is a trigger that launches signaling pathways in MM cells representing an important pathway involved in growth and proliferation (21). Therefore, counteracting IL-6 actions might prove useful not only for the progression of SARS-CoV-2 infection to the acute respiratory distress syndrome, but also for preventing the progression of the underlying Myeloma. Indeed, a recently reported case of MM treated with Tocilizumab showed a favorable outcome, probably due to the timing counteraction of IL-6 receptor by the monoclonal antibody (22). We can assume that, in patients with MM who develop COVID-19 and are not treated, the over-production of IL-6 may play a triggering role capable of unmasking a disease in partial remission and initiating the blast production, as occurred in our patient. Although higher mortality has been reported among patients with uncontrolled MM disease (23), no systematic data exist on the progression of MM during COVID-19. In a recent report, among 650 MM patients with COVID-19, the presence of active or progressive MM (OR = 1.91, 95% CI 0.96–3.81) resulted an independent predictor of adverse outcome on multivariate analysis (24). However, no information is provided regarding the temporal relationship between SARS-CoV-2 infection and disease progression, leaving open the question of whether death is due to SARS-CoV-2 overlap with advanced disease or whether infection favors disease progression thus leading to death. An in deep analysis of inflammatory and immune cell populations and of pro-inflammatory cytokines in MM patients with COVID-19 seems needful to better understand the environment in which MM disease moves during SARS-CoV-2 infection.

Patient #4 had remained on complete remission of her Philadelphia-positive (Ph+) ALL and her CSF-immunophenotypic and cytologic analyses were negative for atypical lymphoid cells until February 2020, when she became SARS-Cov-2 positive with a mild and uneventful clinical course. The infection, in this case, was followed by ALL recurrence within the CNS that coincided with systemic relapse in the marrow and blood. Ph+ ALL is a condition at risk of recurrence, nearly 90% of which occur within three years, and the central nervous system (CNS) is an important site of involvement in adults (25). Indeed, on 27^th^ February, few days before COVID-19 developed, the bone marrow aspirate exhibited a BCR-ABL1/ABL transcript of 0.005, which should be regarded as a risk signal of progression. Nevertheless, the temporal link between SARS-CoV-2 infection and the blast relapse of ALL seems really striking. Therefore, it is intriguing to think that in this Ph+ ALL patient, COVID-19 occurrence has driven a molecular bone marrow relapse with aggressive CNS pattern.

In conclusion, our data represent the first description of hematopoietic disorders involving the B cell lineage, other than hemolytic anemia, occurring or relapsing during acute, proven COVID-19. The temporal relationship of the events may suggest a potential causal relationship between the immunologic and cytokine response to SARS-CoV-2 infection and the hematopoietic disorder. We suggest that special attention should be paid to patients with underlining B cell lineage disorders who acquire COVID-19, possibly exploring the possibility of early counteracting the cytokine storm even in the absence of severe respiratory symptoms.

## Data Availability Statement

The raw data supporting the conclusions of this article will be made available by the authors, without undue reservation.

## Ethics Statement

The studies involving human participants were reviewed and approved by Piacenza Hospital Ethics Committee. The patients/participants provided their written informed consent to participate in this study.

## Members of the COVID-Piacenza Group

Daniela Aschieri, Mario Barbera, Carlo Cagnoni, Luigi Cavanna, Cosimo Franco, Chiara Gorrini, Andrea Magnacavallo, Massimo Nolli, Massimo Piepoli, Roberta Schiavo, Matteo Silva, Marco Stabile, Angela Rossi, Giovanni Vadacca. Affiliations for all members is: “Guglielmo da Saliceto” Piacenza Hospital, Via Taverna 49, 29121 Piacenza, Italy

## Author Contributions

GT made a substantial contribution to the conception and design of the work, to the acquisition, analysis, and interpretation of data for the work, wrote the manuscript, gave final approval of the current version to be published, and agreed to be accountable for all aspects of the work in ensuring that questions related to the accuracy or integrity of any part of the work are appropriately investigated and resolved. DV, EFo, and MC made a substantial contribution to the conception of the work, to the analysis and interpretation of the data for the work, wrote the manuscript, critically revised the manuscript for important intellectual content, gave final approval of the current version to be published, and agreed to be accountable for all aspects of the work in ensuring that questions related to the accuracy or integrity of any part of the work are appropriately investigated and resolved. All other authors gave substantial contributions to the collection and interpretation of the data for the work, revised the manuscript for important intellectual content, gave final approval of the version to be published, and agreed to be accountable for all aspects of the work in ensuring that questions related to the accuracy or integrity of any part of the work are appropriately investigated and resolved. All authors contributed to the article and approved the submitted version.

## Conflict of Interest

The authors declare that the research was conducted in the absence of any commercial or financial relationships that could be construed as a potential conflict of interest.
